# Comparing the effects of biguanides and dipeptidyl peptidase-4 inhibitors on cardio-cerebrovascular outcomes, nephropathy, retinopathy, neuropathy, and treatment costs in diabetic patients

**DOI:** 10.1371/journal.pone.0308734

**Published:** 2024-08-09

**Authors:** Eiji Nakatani, Hiromitsu Ohno, Tatsunori Satoh, Daito Funaki, Chikara Ueki, Taku Matsunaga, Takayoshi Nagahama, Toru Tonoike, Hiromichi Yui, Akinori Miyakoshi, Yoshihiro Tanaka, Ataru Igarashi, Hiraku Kumamaru, Nagato Kuriyama, Akira Sugawara

**Affiliations:** 1 Graduate School of Public Health, Shizuoka Graduate University of Public Health, Shizuoka, Japan; 2 Shizuoka General Hospital, Shizuoka, Japan; 3 Allied Medical K.K., Tokyo, Japan; 4 Institute of Humanistic Social Medicine, Tokyo, Japan; 5 Graduate School of Pharmaceutical Sciences, University of Tokyo, Tokyo, Japan; 6 Graduate School of Data Sciences, Yokohama City University School of Medicine, Yokohama, Japan; 7 Department of Healthcare Quality Assessment, The University of Tokyo, Tokyo, Japan; Keio University School of Medicine, JAPAN

## Abstract

**Background:**

Western guidelines often recommend biguanides as the first-line treatment for diabetes. However, dipeptidyl peptidase-4 (DPP-4) inhibitors, alongside biguanides, are increasingly used as the first-line therapy for type 2 diabetes (T2DM) in Japan. However, there have been few studies comparing the effectiveness of biguanides and DPP-4 inhibitors with respect to diabetes-related complications and cardio-cerebrovascular events over the long term, as well as the costs associated.

**Objective:**

We aimed to compare the outcomes of patients with T2DM who initiate treatment with a biguanide *versus* a DPP-4 inhibitor and the long-term costs associated.

**Methods:**

We performed a cohort study between 2012 and 2021 using a new-user design and the Shizuoka Kokuho database. Patients were included if they were diagnosed with T2DM. The primary outcome was the incidence of cardio-cerebrovascular events or mortality from the initial month of treatment; and the secondary outcomes were the incidences of related complications (nephropathy, renal failure, retinopathy, and peripheral neuropathy) and the daily cost of the drugs used. Individuals who had experienced prior events during the preceding year were excluded, and events within 6 months of the start of the study period were censored. Propensity score matching was performed to compare between two groups.

**Results:**

The matched 1:5 cohort comprised 529 and 2,116 patients who were initially treated with a biguanide or a DPP-4 inhibitor, respectively. Although there were no significant differences in the incidence of cardio-cerebrovascular events or mortality and T2DM-related complications between the two groups (*p* = 0.139 and *p* = 0.595), daily biguanide administration was significantly cheaper (mean daily cost for biguanides, 61.1 JPY; for DPP-4 inhibitors, 122.7 JPY; *p*<0.001).

**Conclusion:**

In patients with T2DM who initiate pharmacotherapy, there were no differences in the long-term incidences of cardio-cerebrovascular events or complications associated with biguanide or DPP-4 use, but the former was less costly.

## Introduction

Type 2 diabetes mellitus (T2DM) is a significant global public health challenge, affecting millions and associated with various complications, particularly cardiovascular and cerebrovascular events [1, 2]. Initial pharmacological interventions for T2DM commonly include biguanides and dipeptidyl peptidase-4 (DPP-4) inhibitors, each with specific mechanisms and potential effects on patient outcomes [3–5]. While Western guidelines often recommend biguanides as the first-line treatment, in Japan, DPP-4 inhibitors alongside biguanides are increasingly used as first-line therapy.

Despite the widespread use of biguanide drugs and DPP-4 inhibitors in the management of diabetes, there is a notable absence of direct comparative studies evaluating their cost-effectiveness in Japan. This gap in the literature underscores the necessity for comprehensive research to assess and compare the economic implications of biguanide drugs and DPP-4 inhibitors within the Japanese healthcare context.

In this study, we compared the long-term effects of initiating treatment with a biguanide or a DPP-4 inhibitor on the incidences of cardiac and cerebrovascular events, diabetic complications, and the associated daily treatment costs in Japanese T2DM patients.

## Materials and methods

### Data source, design, and sample

A retrospective cohort study was performed using a new-user design analysis [6] and the Shizuoka Kokuho Database (SKDB), version 2023 [7], between 1 April 2012 and 30 September 2021. The SKDB contains detailed medical and long-term care insurance data for over 2.3 million individuals, representing a diverse population [7]. Previous studies have leveraged this database to assess the effectiveness and safety of various drugs [8–10]. We accessed the SKDB for research purposes between January 18 and 27, 2024.

The study cohort comprised individuals who were covered by National Health Insurance (aged <75 years) and the Latter-Stage Elderly Medical Care System (aged ≥75 years) in Shizuoka Prefecture, and who had been diagnosed with T2DM. The eligible participants had received a prescription for a first-line diabetes drug following a 1-year baseline health evaluation and a health checkup conducted within the 6 months prior to the initiation of the medication. Patients who had been hospitalized because of acute diabetes mellitus; who had a relevant genetic disease; who had experienced a prior cerebrovascular or cardiac event, cancer, dialysis, glucagon or insulin therapy; or who had undergone home self-injection training during the baseline period were excluded from the analysis.

### First-line treatments

**Fig 1:**
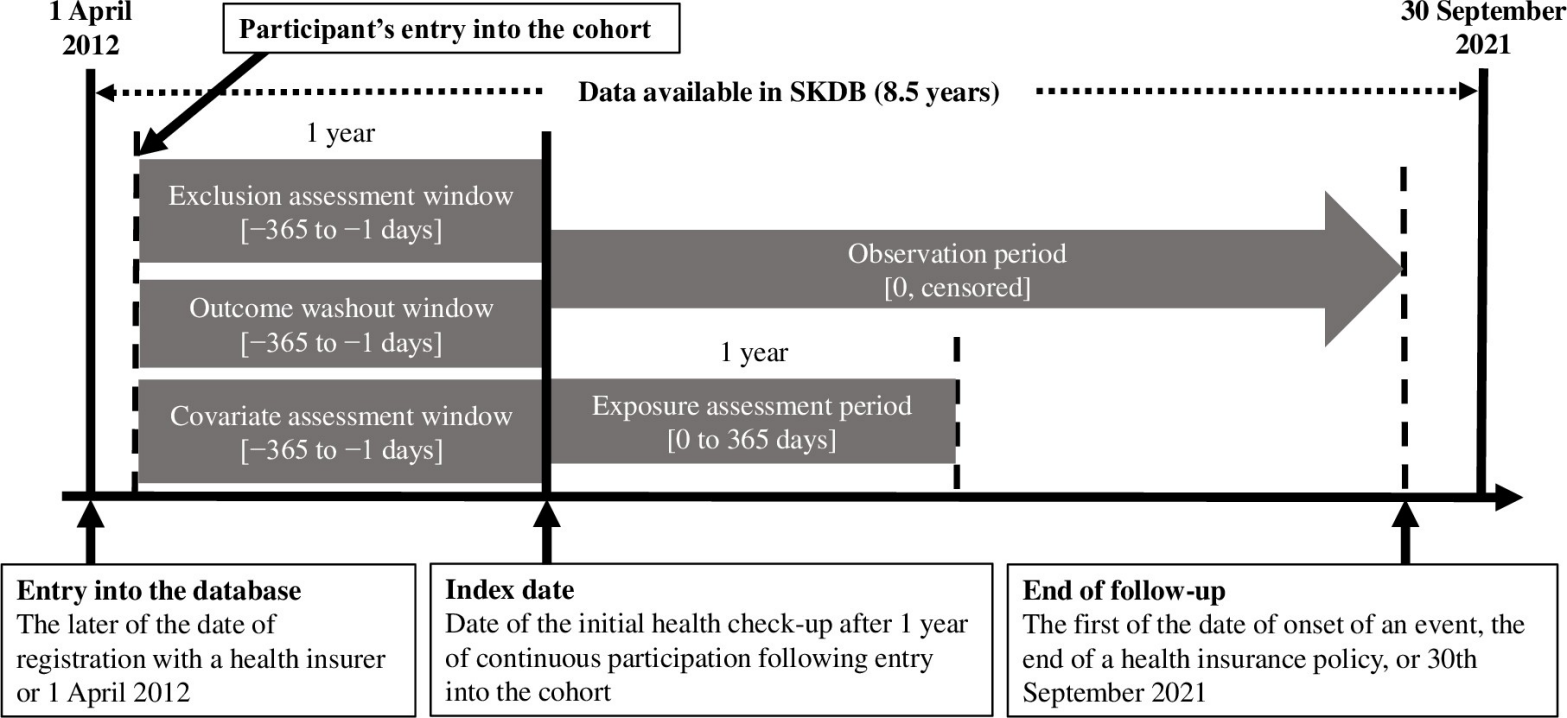
Study schema. DPP-4: dipeptidyl peptidase-4, SKDB: Shizuoka Kokuho Database. Cohort entry was defined as the later of the date of registration with the health insurance provider or April 1, 2012. The index data were collected on the day that the participants were first prescribed a DPP-4 inhibitor or a biguanide. The follow-up period was defined as the interval between the index date and the end date of the study (September 30, 2021), the date of withdrawal from the health insurance system, or the date of occurrence of an outcome.

We compared the effects of an initial prescription of a biguanide alone (metformin hydrochloride or buformin hydrochloride) and a DPP-4 inhibitor alone. In the SKDB, the list of codes used to identify the appropriate patients is shown in S1 Table. The index date for each patient was the date when the initial treatment was prescribed (Fig 1). The patients were analytically allocated to two groups on the basis of the initial treatment administered, which was used as the primary exposure variable. Patients were excluded from the analysis if they did not visit a clinic for a consultation regarding their antidiabetic medication over a period of >6 months within the first year following the index date.

### Potential confounders and other covariates

We made comprehensive adjustments for a range of variables to control for their potential confounding effects on the outcomes. These variables comprised demographic factors, comorbidities, the use of medication, physical activity level, lifestyle habits, and a variety of clinical parameters. Specifically, adjustments were made for sex, age, and comorbid conditions, including hypertension, dementia, cerebrovascular disease, renal disease, rheumatic disease, liver disease, and chronic pulmonary disease. The types of medication that were considered were anti-hypertensive and lipid-lowering agents. Furthermore, the analysis involved adjustment for body mass index (BMI), glycated hemoglobin (HbA1c) level, and lifestyle factors, including the frequency of walking or engaging in physical exercise for more than 1 hour per week, an increase in body mass of >10 kg since the age of 20 years, current smoking status, and heavy alcohol consumption. Heavy alcohol drinking was defined as daily alcohol drinking exceeding 360 mL. Current smokers were defined as those who had smoked over 100 cigarettes or for a minimum of 6 months and were actively smoking for at least the past month. The clinical parameters that were adjusted for comprised gamma-glutamyl transferase (GGT), systolic blood pressure, estimated glomerular filtration rate (eGFR), aspartate aminotransferase (AST), alanine aminotransferase (ALT), low-density lipoprotein (LDL)-cholesterol, triglycerides, and uric acid. The anatomical therapeutic chemical (ATC) codes for the other antidiabetic drugs used for sample selection are listed in S2 Table.

### Outcomes

The primary outcome was the interval between the index date and the earliest of a cerebrovascular event, cardiac event, or mortality, as a composite outcome, defined using the corresponding codes listed in S3 Table. The secondary outcomes were the intervals from the index date to the onset of any diabetes-related event, including diabetic nephropathy, renal failure, diabetic retinopathy, and diabetic peripheral neuropathy, defined using the corresponding codes listed in S4 Table. Participants who were hospitalized or experienced a cerebrovascular event, a cardiac event, cancer, dialysis, or mortality within 6 months of the index date were censored (no event recorded) at that time, such that there was a grace period before the comparison of the effects of the two target drugs.

Cardiac events were defined as hospitalization because of (International Classification of Diseases, 10th Revision) ICD-10 codes I20, I21, I22, I24, I25, and I50, encompassing a range of acute and chronic coronary artery conditions and heart failure, along with the performance of significant interventions, such as percutaneous coronary interventions or coronary artery bypass grafting. This definition aligns with standard clinical and epidemiologic practices in diabetes research and captures the spectrum of cardiovascular complications that are associated with diabetes.

We used the internationally recognized ICD-10 codes (I60, I61, I62, I63, I64) to define stroke and the Japanese health care codes (8838736, 8838748, 8838750) for the identification of other cerebrovascular events. In this way, we aimed to comprehensively and accurately identify cerebrovascular events, while reflecting both international standards and the specific context of Japan.

### Cost per day associated with the use of the antidiabetic drugs

To assess the cost of the medication used by the two treatment groups, the mean daily cost in Japanese Yen (JPY) throughout the observation period was calculated. The advantage of this approach lies in its ability to permit a fair comparison of the costs of treatment over specific periods of time. This method mitigates the effect of differing lengths of observation period by focusing on the mean daily cost, rather than the total cost. Consequently, it enables a more accurate evaluation of the cost-effectiveness of types of medication.

### Statistical analysis

The covariates are summarized using means and standard deviations for continuous data, and frequencies and the corresponding percentages for categorical data. The propensity score for each participant was estimated using a multivariate logistic regression model that incorporated the aforementioned potential confounders. Certain covariates were excluded from the modeling, owing to the difficulty associated with the estimation of their magnitude. We created the cohorts for the analysis by one-to-k matching, based on the propensity scores, with a caliper width set to 0.2. The value of k was determined to be an integer not exceeding 10 following the propensity score matching. Consequently, the size of each study group was maintained at ≥90% of its pre-matching size. The balance of the groups was assessed using standardized mean differences (SMDs), with thresholds set at <10%. For survival time analysis, cumulative incidence curves were plotted. Differences between groups were evaluated using the log-rank test for survival or the composite outcome and Gray’s test [11] for other outcomes, during which mortality was treated as a competing risk. Point estimates and 95% confidence intervals (CIs) for each cumulative incidence were calculated, and hazard ratios (HRs) and the corresponding CIs were also calculated using a cause-specific Cox regression model. To compare the cost per day of the use of the antidiabetic drugs, we used Wilcoxon’s rank-sum test. The mean difference and the 95% CI were estimated using a univariable regression model. As sensitivity analyses for each outcome, the *p*-values obtained using Gray’s test and the HR (95% CI) for the inter-group comparisons for each outcome in participants who attended the clinic for at least 9 months and for the entire 12 months were calculated. Furthermore, subgroup analyses with respect to sex [male, female], age [<70 years, 70–79.9 years, ≥80 years], BMI [<25 kg/m^2^, ≥25 kg/m^2^], hypertension [present, absent], liver disease [present, absent], and the administration of lipid-lowering agents [present, absent], were performed similarly. Simple imputation for missing data was not performed. *P* < 0.05 was interpreted as indicating statistical significance. Statistical analyses were performed using EZR Version 1.61 (Saitama Medical Center, Jichi Medical University, Tochigi, Japan) [12] or SAS Version 9.4 (SAS Institute Inc., Cary, NC, USA).

### Ethics

Our research, which is a retrospective study on data from the SKDB, involved comprehensive anonymization processes before analysis to ensure the privacy and confidentiality of participants [7]. Ethical approval for the study plan (SGUPH_2021_001_078) was granted by the Ethics Committee of Shizuoka Graduate University of Public Health, confirming compliance with all pertinent ethical standards and regulations. Given its retrospective nature and adherence to Japanese medical ethics guidelines, the necessity for informed consent was waived.

## Results

### Characteristics of the participants

**Fig 2:**
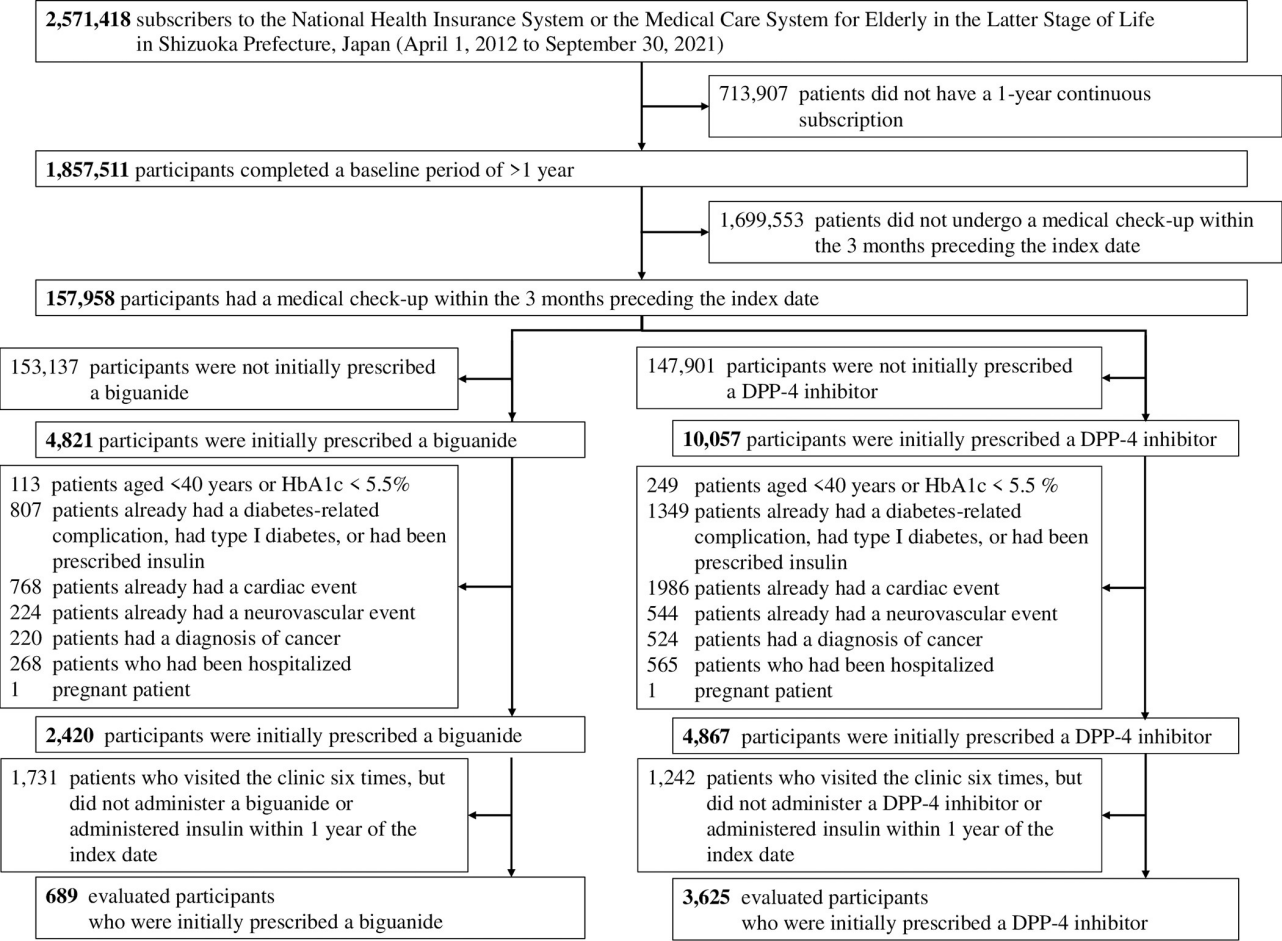
Flow diagram for the participants.

A flow chart describing the sample selection is shown in Fig 2. Comparisons of the characteristics of the participants before and after propensity matching are shown in S5 Table and Table 1, respectively. Before propensity score matching, there were differences in the ages of the participants when the drug was first prescribed, the prevalence of dementia, BMI during a medical checkup, and HbA1c between the biguanide and DPP-4 inhibitor groups (SMD > 0.15, S5 Table). However, matching was conducted based on the characteristics of the biguanide group, such that the initial distributions of all these variables were aligned, making the two groups comparable (SMD < 0.1, Table 1).

**Table 1:** Participant characteristics after propensity score matching.

Variable	Category (unit)	After matching		SMD
		Biguanide	DPP-4 inhibitor	
		N = 514	N = 2,570	
Sex	Male	239 (46.5)	1206 (46.9)	0.009
Age	(years)	68.39 (7.59)	68.67 (7.58)	0.036
	40 to 49.9 years	13 (2.5)	56 (2.2)	0.074
	50 to 59.9 years	40 (7.8)	174 (6.8)	
	60 to 69.9 years	226 (44.0)	1156 (45.0)	
	70 to 79.9 years	204 (39.7)	992 (38.6)	
	≥80 years	31 (6.0)	192 (7.5)	
**Comorbidities**				
Hypertension	Presence	297 (57.8)	1498 (58.3)	0.010
Dementia	Presence	3 (0.6)	8 (0.3)	0.041
Cerebrovascular disease	Presence	30 (5.8)	159 (6.2)	0.015
Renal disease	Presence	6 (1.2)	35 (1.4)	0.017
Rheumatic disease	Presence	10 (1.9)	55 (2.1)	0.014
Liver disease	Presence	115 (22.4)	499 (19.4)	0.073
Chronic pulmonary disease	Presence	87 (16.9)	444 (17.3)	0.009
**Medication**				
Anti-hypertensive agent	Yes	255 (49.6)	1386 (53.9)	0.087
Lipid-lowering agent	Yes	249 (48.4)	1196 (46.5)	0.038
**Medical checkup**				
BMI	(kg/m^2^)	24.72 (3.67)	24.67 (3.88)	0.013
	<18.50 kg/m^2^	19 (3.7)	77 (3.0)	0.073
	18.50 to 21.99 kg/m^2^	95 (18.5)	533 (20.7)	
	22.00 to 24.99 kg/m^2^	177 (34.4)	848 (33.0)	
	25.00 to 29.99 kg/m^2^	182 (35.4)	891 (34.7)	
	≥30.00 kg/m^2^	41 (8.0)	221 (8.6)	
HbA1c	(%)	7.24 (1.17)	7.22 (1.15)	0.017
	<6.00%	13 (2.5)	82 (3.2)	0.062
	6.00 to 6.49%	84 (16.3)	444 (17.3)	
	6.50 to 6.99%	149 (29.0)	753 (29.3)	
	7.00 to 7.99%	188 (36.6)	879 (34.2)	
	≥8.00%	80 (15.6)	412 (16.0)	
Walking or physical exercise for >1 hour/week	Yes	193 (43.9)	923 (44.5)	0.013
Current smoker	Yes	65 (12.6)	363 (14.1)	0.043
Heavy alcohol drinking	Yes	27 (5.3)	130 (5.1)	0.009
GGT	(U/L)	49.29 (72.11)	46.51 (55.21)	0.043
Systolic blood pressure	(mmHg)	133.01 (16.39)	133.68 (16.73)	0.041
Estimated GFR	(mL/min/1.73 m^2^)	72.40 (15.18)	72.32 (16.47)	0.005
AST	(U/L)	30.22 (22.08)	29.18 (24.04)	0.045
ALT	(U/L)	27.96 (15.12)	27.32 (15.49)	0.042
LDL-cholesterol	(mg/dL)	127.08 (31.43)	128.26 (32.28)	0.037
Triglycerides	(mg/dL)	149.02 (122.46)	145.59 (99.44)	0.031
Uric acid	(mg/dL)	5.28 (1.28)	5.29 (1.28)	0.007

ALT, alanine aminotransferase; AST, aspartate aminotransferase; BMI, body mass index; DPP-4, dipeptidyl peptidase 4; GFR, glomerular filtration rate; GGT, gamma-glutamyl transpeptidase; HbA1c, hemoglobin A1c; LDL, low-density lipoprotein; SMD: standardized mean difference.

### Prescriptions of antidiabetic medication and clinic visits following propensity score matching

The antidiabetic medication being used and the number of clinic visits made prior to matching are shown in S6 Table. Table 2 shows the antidiabetic medication prescriptions and the number of healthcare visits within the year following the index date and propensity score matching for participants who were treated with a biguanide (n = 514) or a DPP-4 inhibitor (n = 2,570). After matching, 100% of the participants remained on their respective initial treatments (a biguanide or a DPP-4 inhibitor), and none were administering insulin. Very few participants were being prescribed a GLP-1 receptor agonist (0.2% in the biguanide group and 0.1% in the DPP-4 inhibitor group). SGLT2 inhibitors were more commonly prescribed in the biguanide group (9.3%) than in the DPP-4 inhibitor group (4.6%). The prevalences of alpha-glucosidase inhibitor and thiazolidinedione administration also slightly differed between the two groups. Rapid-acting secretagogues were equally frequently prescribed in the two groups (2.5%), but sulfonylureas were slightly more frequently prescribed to the DPP-4 inhibitor group (12.1% *vs*. 10.3% for the biguanide group). The mean number of healthcare visits was similar for the two groups, with the biguanide group averaging 10.68 visits per year and the DPP-4 inhibitor group averaging 10.80 visits, indicating comparable levels of healthcare engagement following matching.

**Table 2:** Antidiabetic medication prescribed and the number of health care visits within the year following the index date and after propensity score matching.

Variable	After matching	
	Biguanide (n = 514)	DPP-4 inhibitor (n = 2,570)
Biguanides	514 (100.0)	0
DPP-4 inhibitors	0	2,570 (100.0)
Insulin	0	0
GLP-1 receptor agonists	1 (0.2)	2 (0.1)
SGLT2 inhibitors	48 (9.3)	117 (4.6)
Alpha-glucosidase inhibitors	46 (8.9)	206 (8.0)
Thiazolidinediones (also known as glitazones)	25 (4.9)	92 (3.6)
Rapid-acting secretagogues (meglitinides, also known as glinides)	13 (2.5)	63 (2.5)
Sulfonylureas	53 (10.3)	311 (12.1)
Number of healthcare visits (month, per year)	10.68 (1.80)	10.80 (1.68)

DPP-4, dipeptidyl peptidase 4; GLP, glucagon-like peptide-1; SGLT2, sodium–glucose cotransporter 2.

### Cardiac and cerebrovascular outcomes

**Fig 3:**
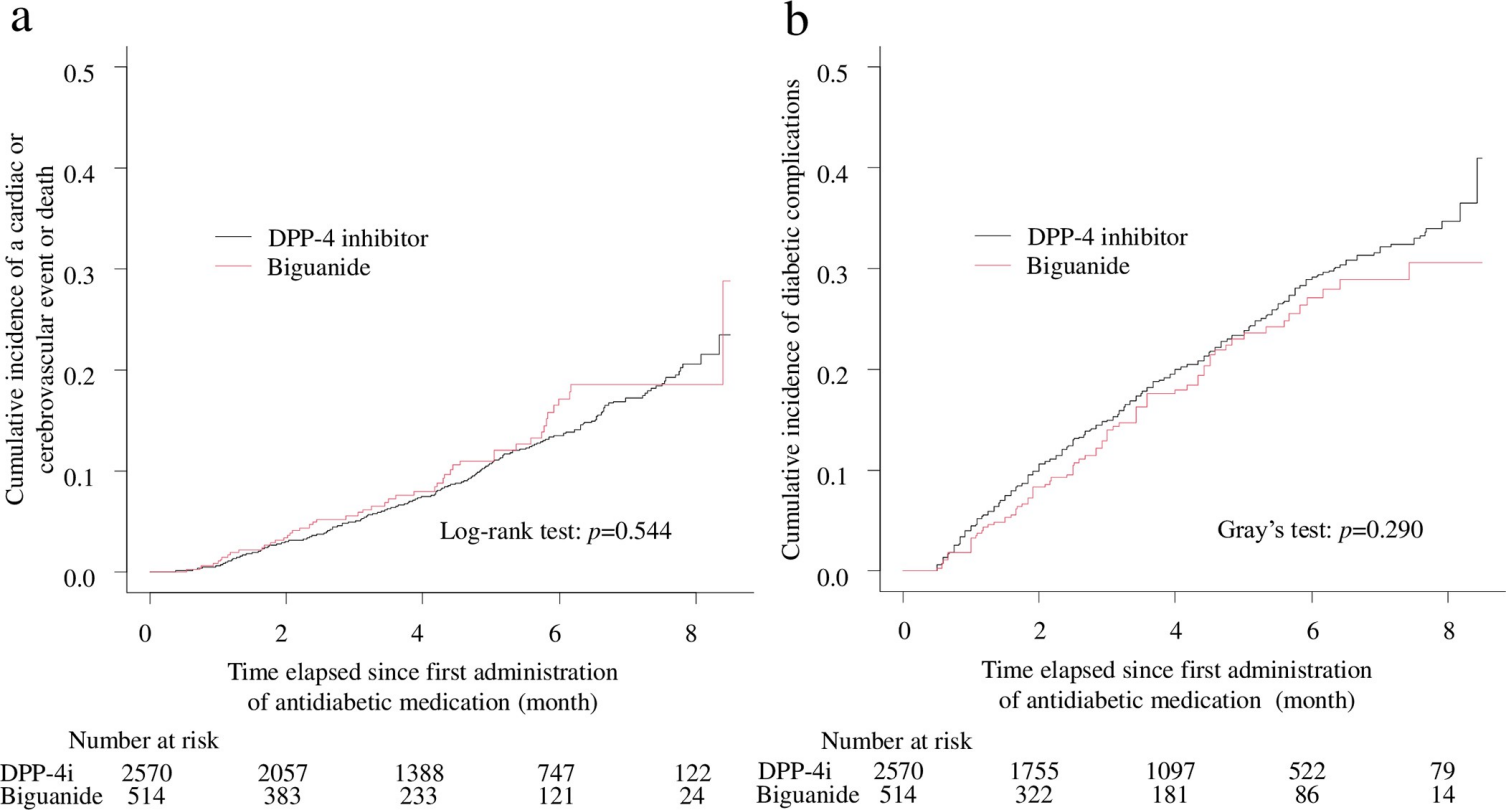
Cumulative incidences of cardiac and cerebrovascular events or mortality, and diabetic complications in the matched cohort. DPP-4i: dipeptidyl peptidase-4 inhibitor. Cumulative incidences of (a) the composite outcome of cardiac or cerebrovascular outcomes or mortality and (a) diabetic complications, including diabetic retinopathy, nephropathy, neuropathy, and other conditions, in the propensity score-matched cohort.

The matched cohort consisted of 514 participants who were prescribed biguanides and 2,570 who were prescribed DPP-4 inhibitors during the observation period (1:5 matching ratio; median 4.0 years, maximum 8.5 years). Cardiac events occurred in 23 participants (4.5%) in the biguanide group and 149 participants (5.8%) in the DPP-4 inhibitor group. Cerebrovascular events occurred in 17 participants (3.3%) in the biguanide group and 84 participants (3.3%) in the DPP-4 inhibitor group. There were 16 deaths (3.1%) in the biguanide group and 91 (3.5%) in the DPP-4 inhibitor group. Therefore, the composite outcome occurred in 49 participants (9.5%) in the biguanide group and 267 participants (10.4%) in the DPP-4 inhibitor group (Table 3). The incidence of the cardio-cerebrovascular composite outcome did not significantly differ between the groups (*p* = 0.544; Table 3 and Fig 3A). The HR for the biguanide *vs*. DPP-4 inhibitor group was 1.06 (95% CI: 0.79–1.44), indicating no significant difference.

**Table 3:** Comparison of the outcomes of participants who were prescribed biguanide or a DPP-4 inhibitor.

Outcome	Exposure	Events, number (%)	Cumulative incidence after 5 years		*P*-value
			Rate	95% confidence interval	
Composite event^†^	Biguanide (n = 514)	49 (9.5)	11.0	8.0–15.1	0.544
	DPP-4 inhibitor (n = 2,570)	267 (10.4)	10.8	9.4–12.4	
Cardiac event*	Biguanide (n = 514)	23 (4.5)	5.2	3.1–8.1	0.825
	DPP-4 inhibitor (n = 2,570)	149 (5.8)	6.3	5.2–7.5	
Cerebrovascular event*	Biguanide (n = 514)	17 (3.3)	3.9	2.2–6.4	0.538
	DPP-4 inhibitor (n = 2,570)	84 (3.3)	3.1	2.4–4.0	
Death^†^	Biguanide (n = 514)	16 (3.1)	3.3	1.7–6.1	0.860
	DPP-4 inhibitor (n = 2,570)	91 (3.5)	3.7	2.8–4.7	
Diabetic complication*	Biguanide (n = 514)	79 (15.4)	23.3	18.5–28.5	0.290
	DPP-4 inhibitor (n = 2,570)	439 (17.1)	22.9	20.9–24.9	
Diabetic retinopathy*	Biguanide (n = 514)	60 (11.7)	16.3	12.3–20.7	0.602
	DPP-4 inhibitor (n = 2,570)	374 (14.6)	16.9	15.2–18.7	
Diabetic nephropathy*	Biguanide (n = 514)	21 (4.1)	6.2	3.8–9.3	0.983
	DPP-4 inhibitor (n = 2,570)	118 (4.6)	4.9	4.0–5.9	
Diabetic neuropathy*	Biguanide (n = 514)	7 (1.4)	2.0	0.8–4.3	0.560
	DPP-4 inhibitor (n = 2,570)	50 (2.0)	1.7	1.2–2.3	
Other conditions*	Biguanide (n = 514)	14 (2.7)	2.6	1.2–4.7	0.790
	DPP-4 inhibitor (n = 2,570)	75 (2.9)	3.1	2.4–4.0	

*Gray’s test was performed

^†^The log-rank test was performed. DPP-4: dipeptidyl peptidase 4.

**Fig 4:**
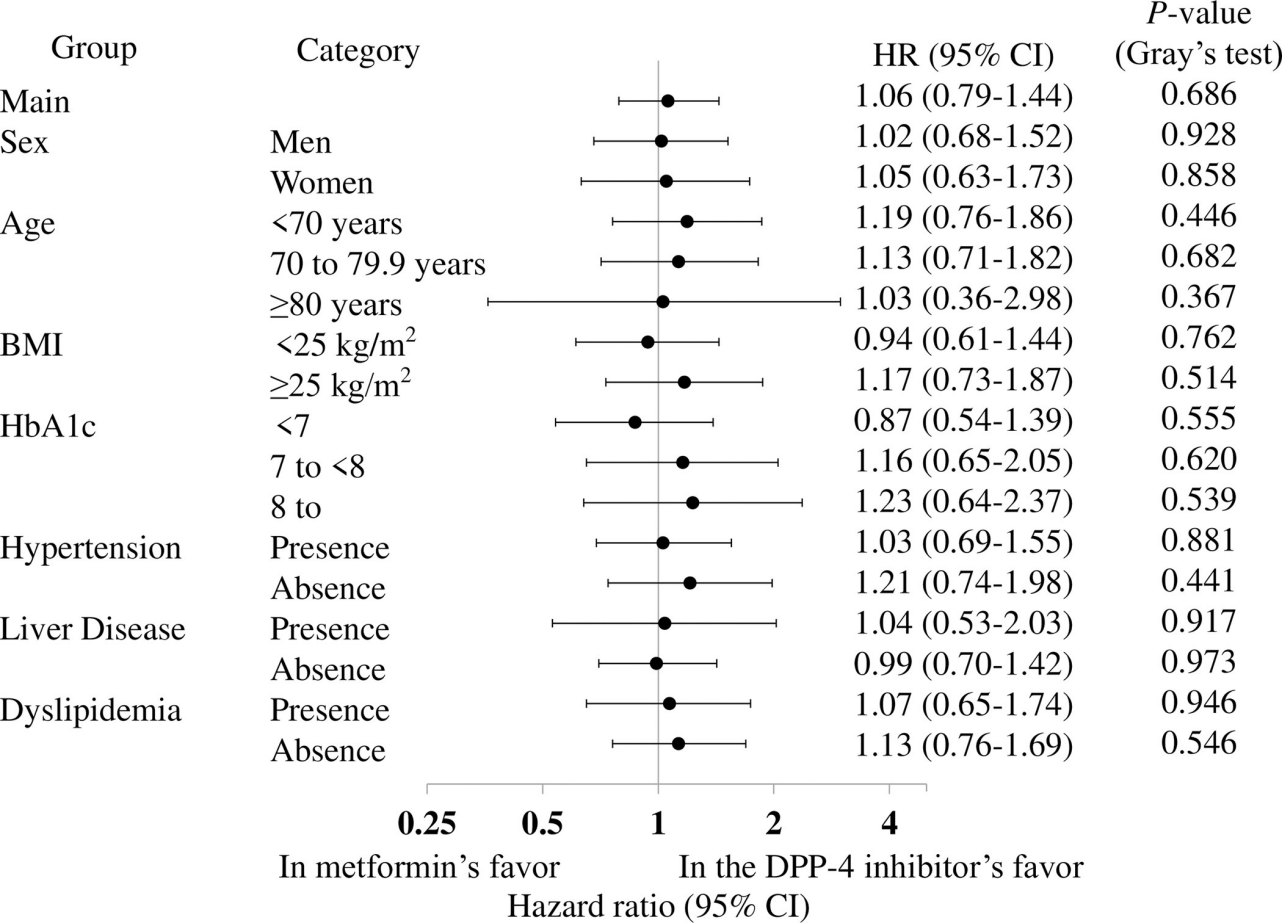
Results of the subgroup analysis of the composite outcome. HR: hazard ratio, CI: confidence interval.

With respect to the sensitivity analyses, there were no differences in the incidences of the composite outcome based on population with visits of 9- and 12-months made during the 12 months following the index date (*p* = 0.395 and *p* = 0.835, respectively; S7 and S8 Tables). Furthermore, the subgroup analyses also showed no significant differences (Fig 4).

### Diabetic complications

During the observation period, diabetic complications occurred in 79 participants (15.4%) in the biguanide group and 439 participants (17.1%) in the DPP-4 inhibitor group (*p* = 0.290; Table 3 and Fig 3B). The cumulative incidence of diabetic retinopathy, nephropathy, neuropathy, and other conditions between the two groups also indicated no significant difference (*p* = 0.579, *p* = 0.894, *p* = 0.491, and *p* = 0.891; Table 3).

The HR of biguanide use *vs*. DPP-4 inhibitor use for diabetic complications was 0.88 (95% CI: 0.70–1.13), indicating no significant difference. The HRs of biguanide use *vs*. DPP-4 inhibitor use for diabetic retinopathy, nephropathy, neuropathy, and other conditions were 0.93 (0.71–1.22), 0.97 (0.61–1.54), 0.76 (0.34–1.67), and 1.04 (0.59–1.84), indicating no significant difference.

**Fig 5:**
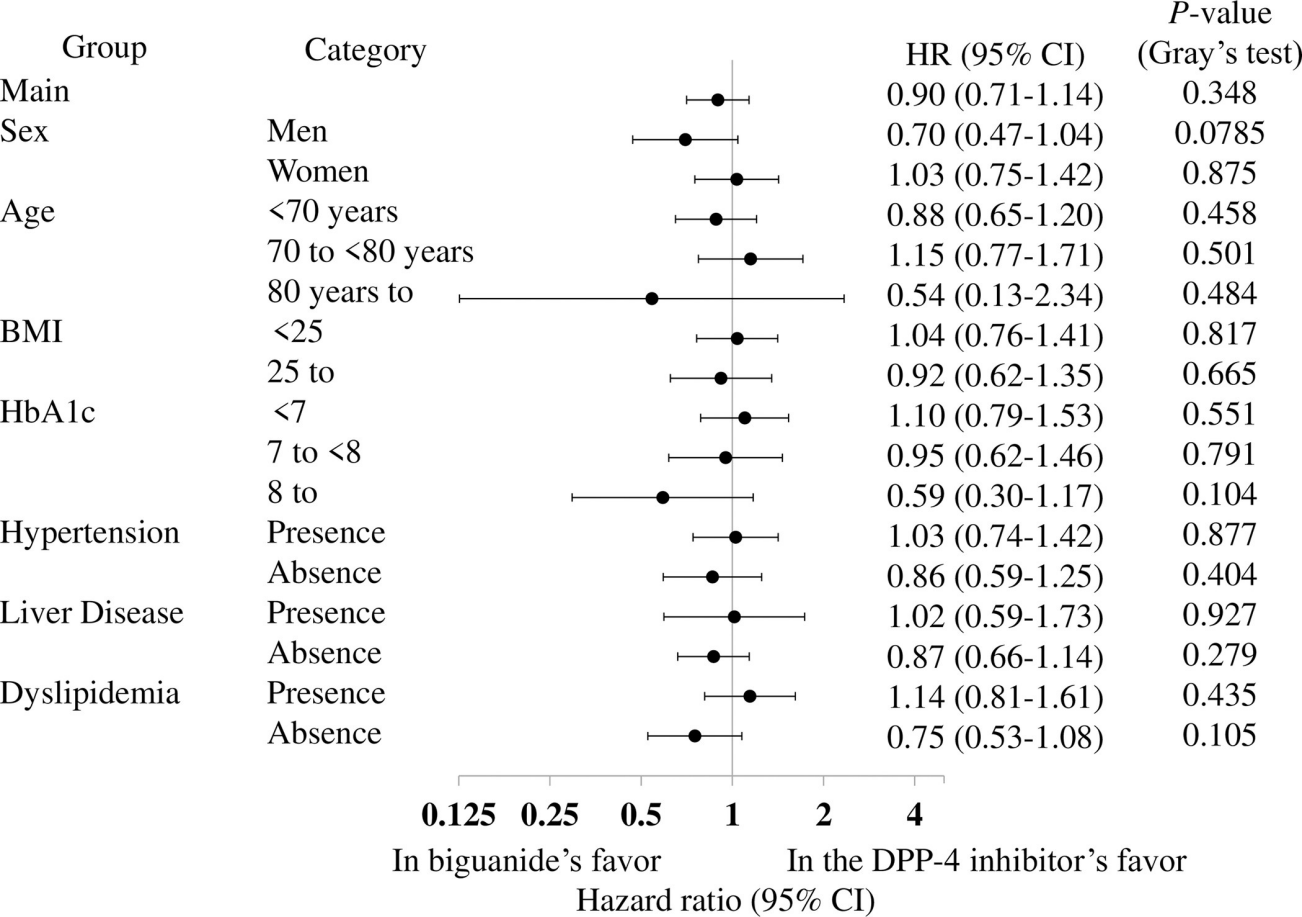
Results of the subgroup analysis of diabetic complications. HR: hazard ratio, CI: confidence interval.

The sensitivity analyses indicated that the incidences of the composite outcomes for diabetic complications did not differ after 9 or 12 months, based on population with visits of 9- and 12-months made during the 12 months following the index date (*p* = 0.722 and *p* = 0.348, respectively; S7 and S8 Tables). Furthermore, the results of the subgroup analyses, as shown in Fig 5, showed no significant differences.

### Daily cost of the antidiabetic medication

The mean daily costs of the antidiabetic drug use were 60.5 ± 70.9 yen for biguanides and 123.6 ± 64.3 yen for DPP-4 inhibitors, with a mean difference (95% CI) of 63.1 (56.9–69.3) yen. Thus, the cost of using a biguanide was lower (*p* < 0.001).

## Discussion

In our study comparing the effectiveness and cost of DPP-4 inhibitors and biguanides in Japanese T2DM patients, we found no significant differences in long-term incidences of cardio-cerebrovascular events and complications between the two groups, but the daily cost for antidiabetic agents of biguanide use was lower.

Metformin is celebrated for its effectiveness, safety, and financial benefits; it primarily reduces hepatic glucose production and enhances insulin sensitivity, lowering both fasting and postprandial blood glucose levels [13]. Unlike many antidiabetic agents, metformin does not promote weight gain or significantly increase the risk of hypoglycemia, making it the preferred first-line therapy in Japan [14]. Additionally, metformin may have cardiovascular benefits, reduce cancer risk [15, 16], and lower the risk of cognitive decline with long-term use [17]. These findings underscore the importance of considering cost alongside clinical effectiveness when selecting initial treatments for T2DM, particularly in light of rising healthcare costs. Opting for less expensive treatments with similar effectiveness can reduce the financial burden on patients without compromising care. Previous studies, including a systematic review [18], have focused on the general effectiveness and safety of biguanides and DDP-4 inhibitors in T2DM management. We add to this knowledge by evaluating their cost-effectiveness, an often-overlooked aspect crucial for healthcare decision-making. Demonstrating the economic benefits of biguanides, our study provides a comprehensive assessment of potential treatment strategies and enriches the dialogue surrounding diabetes management.

DPP-4 inhibitors are important for T2DM treatment and have a favorable safety profile for many patients [19]. They are crucial in diabetes management by preventing incretin hormone degradation and enhancing insulin secretion, effectively managing postprandial hyperglycemia [20, 21]. Their widespread use in Japan to improve glycemic control in T2DM patients, along with their mild side effects, demonstrates their high regard [22]. We found no significant differences in cardiovascular events or diabetes complications between patients treated with DPP-4 inhibitors and biguanides. This aligns with findings that DPP-4 inhibitors reduce hypoglycemia risk when combined with sulfonylureas [23] and are as effective as GLP-1 analogs. Their safety is demonstrated in patients with high cardiovascular risk and renal impairment [5]. DPP-4 inhibitors improve glycemic control without causing weight gain, maintaining a benign adverse event profile [24]. They balance safety and effectiveness in T2DM treatment and are preferred by Japanese patients for treatment satisfaction [25]. However, they may be associated with higher risks of adverse events such as asthenia, cardiac, and vascular disorders [26], and heart failure [27]. Moreover, geographic differences exist in adverse outcomes, with studies in Taiwan, Hong Kong, and the United States showing higher incidences of heart failure, hyperlipidemia, and renal failure with DPP-4 inhibitors compared to biguanides [28]. Additionally, DPP-4 inhibitors are associated with a higher risk of infections, especially upper respiratory tract infections [29]. Despite these risks, DPP-4 inhibitors have advantages like lower hypoglycemia risk and weight management benefits [30, 31]. Specific DPP-4 inhibitors, such as vildagliptin and sitagliptin, are beneficial for older patients due to their tolerability and low incidence of gastrointestinal side effects, and they are effective and safe for patients with renal impairment [32, 33]. However, given the significant cost advantage of biguanides, prescribing behaviors should be reevaluated considering the higher unit costs of DPP-4 inhibitors and the long-term safety, effectiveness, and lower cost of metformin.

Previous research also found the annual cost of DPP-4 inhibitors significantly higher [34]. Despite this, DPP-4 inhibitors can be cost-effective for patients whose glycemia cannot be controlled with metformin alone [35]. For T2DM patients who do not achieve glycemic targets with metformin alone, our findings suggest that a DPP-4 inhibitor is a viable alternative, reducing HbA1c similarly to sulfonylurea or pioglitazone without affecting body mass [18]. Therefore, the financial implications of choosing a DPP-4 inhibitor over a biguanide must be carefully considered in Japanese T2DM patients.

The study had several limitations. First, the SKDB-based methodology affects the generalizability of the results. Second, the geographic specificity of the database limits its applicability to other regions or demographics. Third, retrospective cohort studies carry risks of bias, especially from unmeasured confounders, making causal links difficult to establish. Fourth, selection bias from the exclusion criteria may limit applicability to patients with severe conditions or specific comorbidities. Fifth, despite using propensity score matching, unmeasured variables like dietary habits and physical activity might have influenced the outcomes. Future studies should evaluate these factors through detailed patient surveys or longitudinal monitoring. Sixth, conclusions based on Japanese patient data may not generalize to other ethnicities due to genetic and physiological differences affecting drug metabolism and effectiveness. Although we did not analyze ethnicity effects, such differences could lead to significant variations. Seventh, we did not include changes in HbA1c as an outcome due to inconsistent data availability in the SKDB. HbA1c data was only available for patients who underwent health check-ups in subsequent years, which limited our ability to accurately track post-baseline HbA1c levels. Last, using ICD-10 codes to determine outcomes may not capture all early diabetic complications such as microalbuminuria or unmedicated diabetic neuropathy, potentially underestimating their true incidence.

## Conclusions

In patients with T2DM who commence pharmacotherapy, biguanides are associated with similar incidences of long-term cardio-cerebrovascular events and complications to DPP-4 inhibitors, but are cheaper. These findings underscore the therapeutic effectiveness and financial benefits of administering biguanides as the initial treatment for T2DM.

## Supporting information

S1 Table Search codes for DPP-4 inhibitors and biguanides. DPP-4: dipeptidyl peptidase-4.
(DOCX)

S2 Table ATC codes for other antidiabetic medication. ATC: anatomical therapeutic chemical, DPP-4: dipeptidyl peptidase-4, GLP-1: glucagon-like peptide-1, SGLT2: Sodium–glucose cotransporter 2.
(DOCX)

S3 Table Definitions of outcomes. ICD-10: International Classification of Diseases, 10th Revision.
(DOCX)

S4 Table Diagnostic codes for diabetes in Japan. ICD-10: International Classification of Diseases, 10th Revision.
(DOCX)

S5 Table Characteristics of the participants prior to propensity score matching. ALT, alanine aminotransferase; AST, aspartate aminotransferase; BMI, body mass index; DPP-4, dipeptidyl peptidase 4; GFR, glomerular filtration rate; GGT, gamma-glutamyl transpeptidase; HbA1c, hemoglobin A1c; LDL, low-density lipoprotein; SMD: standardized mean difference.
(DOCX)

S6 Table Antidiabetic medication prescribed and the number of health care visits within the year following the index date, before propensity score matching. DPP-4: dipeptidyl peptidase-4; GLP-1: glucagon-like peptide-1; SGLT2: sodium–glucose cotransporter 2.
(DOCX)

S7 Table Outcomes of the participants who were prescribed biguanide or a DPP-4 inhibitor in the matched cohort and who attended the clinic for ≥9 months, as a sensitivity analysis (n = 2,634). *Gray’s test was performed. ^†^The log-rank test was performed. DPP-4: dipeptidyl peptidase 4 inhibitor.
(DOCX)

S8 Table Outcomes of participants who were prescribed biguanide or a DPP-4 inhibitor in the matched cohort and who had attended the clinic for 12 months, as a sensitivity analysis (n = 1,602). *Gray’s test was performed. ^†^The log-rank test was performed. DPP-4: dipeptidyl peptidase 4 inhibitor.
(DOCX)
