# Supplementary material for: Comparing the effects of biguanides and dipeptidyl peptidase-4 inhibitors on cardio-cerebrovascular outcomes, nephropathy, retinopathy, neuropathy, and treatment costs in diabetic patients
Source: PLoS One. 2024 Aug 9;19(8):e0308734. doi: 10.1371/journal.pone.0308734 (PMC11315305; doi:10.1371/journal.pone.0308734)
Supplement: S1 Table — DPP-4: dipeptidyl peptidase-4. (DOCX) [file pone.0308734.s001.docx]

**S1 Table.** Search codes for DPP-4 inhibitors and biguanides.

| **Medication** | **Code** |
| --- | --- |
| **DPP-4 inhibitor/combination** |  |
| Alogliptin benzoate | 621986001, 621986101, 621986201 |
| Alogliptin benzoate + Metformin hydrochloride | 622517101 |
| Alogliptin benzoate + Pioglitazone hydrochloride | 622086001, 622086101 |
| Anagliptin | 622201701 |
| Anagliptin + Metformin hydrochloride | 622654401, 622654501 |
| Linagliptin | 622093501 |
| Omarigliptin | 622448901, 622449001 |
| Saxagliptin hydrate | 622245601, 622245701 |
| Sitagliptin phosphate hydrate | 621950901, 621951001, 621951101, 621970601, 621970701, 621970801, 622277501, 622288401 |
| Teneligliptin hydrobromide hydrate | 622182601, 622660601, 622861201, 622861301 |
| Trelagliptin succinate | 622415401, 622415501, 622699501 |
| Vildagliptin | 621980701 |
| Vildagliptin + Metformin hydrochloride | 622450301, 622450401 |
| **Biguanides** |  |
| Buformin hydrochloride | 620004502, 620005979, 620873901 |
| Metformin hydrochloride | 610444147, 610463145, 620002859, 620004480, 620005570, 621676001, 621974701, 622070801, 622242501, 622412701, 622417101, 622417201, 622421101, 622421201, 622421901, 622422001, 622424401, 622424501, 622427201, 622427301, 622432601, 622432701, 622436301, 622438401, 622438501, 622448601, 622466601, 622784601, 622784701, 622822401, 622822501 |

DPP-4: dipeptidyl peptidase-4.
