# Supplementary material for: Comparing the effects of biguanides and dipeptidyl peptidase-4 inhibitors on cardio-cerebrovascular outcomes, nephropathy, retinopathy, neuropathy, and treatment costs in diabetic patients
Source: PLoS One. 2024 Aug 9;19(8):e0308734. doi: 10.1371/journal.pone.0308734 (PMC11315305; doi:10.1371/journal.pone.0308734)
Supplement: S2 Table — ATC: anatomical therapeutic chemical, DPP-4: dipeptidyl peptidase-4, GLP-1: glucagon-like peptide-1, SGLT2: Sodium–glucose cotransporter 2. (DOCX) [file pone.0308734.s002.docx]

**S2 Table.** ATC codes for other antidiabetic medications.

| **ATC code** | **Drug class** |
| --- | --- |
| A10B | GLP-1 receptor agonists |
| A10C | Insulin |
| A10H | Sulfonylureas |
| A10J | Biguanides |
| A10K | Glitazones |
| A10L | Alpha-glucosidase inhibitors |
| A10M | Glinides |
| A10N | DPP-4 inhibitors |
| A10P | SGLT2 inhibitors |
| A10X | Other antidiabetic medication |

ATC: anatomical therapeutic chemical, DPP-4: dipeptidyl peptidase-4, GLP-1: glucagon-like peptide-1, SGLT2: Sodium–glucose cotransporter 2.
