# Supplementary material for: Comparing the effects of biguanides and dipeptidyl peptidase-4 inhibitors on cardio-cerebrovascular outcomes, nephropathy, retinopathy, neuropathy, and treatment costs in diabetic patients
Source: PLoS One. 2024 Aug 9;19(8):e0308734. doi: 10.1371/journal.pone.0308734 (PMC11315305; doi:10.1371/journal.pone.0308734)
Supplement: S3 Table — ICD-10: International Classification of Diseases, 10th Revision. (DOCX) [file pone.0308734.s003.docx]

**S3 Table.** Definitions of outcomes.

| **Outcome** | **ICD-10 or disease code** | **Detail** |
| --- | --- | --- |
| Cardiac event | I20, I21, I22, I24 as acute coronary syndromes  I25 as chronic heart disease  I50 as heart failure | Of these coded participants, only those who were hospitalized were considered to have experienced events |
|  | 150260350, 150284310, 150318310, 150359310,  150153910,150374910, 150375010, 150375110,  150263310, 150375210, 150375310, 150375410,  150145910, 150146010, 150318410, 150318510 | Percutaneous coronary intervention or coronary artery bypass grafting |
| Cerebrovascular event | I60, I61, I62, I63, I64 as stroke  8838736, 8838748, 8838750 as another cerebrovascular event | Of these coded patients, only those who were hospitalized were considered to have experienced events |

ICD-10: International Classification of Diseases, 10th Revision.
