# Supplementary material for: Comparing the effects of biguanides and dipeptidyl peptidase-4 inhibitors on cardio-cerebrovascular outcomes, nephropathy, retinopathy, neuropathy, and treatment costs in diabetic patients
Source: PLoS One. 2024 Aug 9;19(8):e0308734. doi: 10.1371/journal.pone.0308734 (PMC11315305; doi:10.1371/journal.pone.0308734)
Supplement: S5 Table — ALT, alanine aminotransferase; AST, aspartate aminotransferase; BMI, body mass index; DPP-4, dipeptidyl peptidase 4; GFR, glomerular filtration rate; GGT, gamma-glutamyl transpeptidase; HbA1c, hemoglobin A1c; LDL, low-density lipoprotein; SMD: standardized mean difference. (DOCX) [file pone.0308734.s005.docx]

**S5 Table.** Characteristics of the participants prior to propensity score matching.

| **Variable** | **Category**  **(unit)** | **Before matching** | | **SMD** |
| --- | --- | --- | --- | --- |
|  |  | **Biguanide** | **DPP-4 inhibitor** |  |
|  |  | **N=689** | **N=3,625** |  |
| Sex | Male | 338 (49.1) | 1686 (46.5) | 0.051 |
| Age | (year) | 66.85 (8.36) | 70.29 (8.21) | 0.415 |
|  | 40 to 49.9 years | 72 (2.0) | 35 (5.1) | 0.410 |
|  | 50 to 59.9 years | 197 (5.4) | 69 (10.0) |  |
|  | 60 to 69.9 years | 1389 (38.3) | 320 (46.4) |  |
|  | 70 to 79.9 years | 1484 (40.9) | 231 (33.5) |  |
|  | ≥80 years | 483 (13.3) | 34 (4.9) |  |
| **Comorbidities** |  |  |  |  |
| Hypertension | Presence | 387 (56.2) | 2140 (59.0) | 0.058 |
| Dementia | Presence | 3 (0.4) | 78 (2.2) | 0.152 |
| Cerebrovascular disease | Presence | 44 (5.7) | 282 (7.3) | 0.062 |
| Renal disease | Presence | 8 (1.0) | 55 (1.4) | 0.034 |
| Rheumatic disease | Presence | 18 (2.3) | 77 (2.0) | 0.025 |
| Liver disease | Presence | 174 (22.6) | 769 (19.8) | 0.070 |
| Chronic pulmonary disease | Presence | 123 (16.0) | 674 (17.3) | 0.036 |
| **Medication** |  |  |  |  |
| Anti-hypertensive agent | Yes | 373 (48.5) | 2080 (53.5) | 0.100 |
| Lipid-lowering agent | Yes | 351 (45.6) | 1762 (45.3) | 0.006 |
| **Medical checkup** |  |  |  |  |
| BMI | (kg/m^2^) | 25.39 (4.19) | 24.33 (3.82) | 0.266 |
|  | <18.50 kg/m^2^ | 20 (2.6) | 148 (3.8) | 0.239 |
|  | 18.50 to 21.99 kg/m^2^ | 123 (16.0) | 883 (22.7) |  |
|  | 22.00 to 24.99 kg/m^2^ | 253 (32.9) | 1345 (34.6) |  |
|  | 25.00 to 29.99 kg/m^2^ | 283 (36.8) | 1222 (31.4) |  |
|  | ≥30.00 kg/m^2^ | 90 (11.7) | 288 (7.4) |  |
| HbA1c | (%) | 7.36 (1.32) | 7.20 (1.11) | 0.135 |
|  | <6.00% | 26 (3.4) | 112 (2.9) | 0.194 |
|  | 6.00% to 6.49% | 120 (15.6) | 617 (15.9) |  |
|  | 6.50% to 6.99% | 209 (27.2) | 1250 (32.2) |  |
|  | 7.00% to 7.99% | 254 (33.0) | 1364 (35.1) |  |
|  | ≥8.00% | 160 (20.8) | 544 (14.0) |  |
| Walking or physical exercise for >1 hour/week | Yes | 248 (41.8) | 1303 (45.4) | 0.073 |
| Current smoker | Yes | 104 (15.1) | 467 (12.9) | 0.064 |
| Heavy alcohol drinking | Yes | 36 (5.2) | 174 (4.8) | 0.019 |
| GGT | (U/L) | 49.32 (65.20) | 46.83 (62.07) | 0.039 |
| Systolic blood pressure | (mmHg) | 132.78 (16.47) | 134.29 (16.86) | 0.090 |
| Estimated GFR | (mL/min/1.73 m^2^) | 73.40 (15.26) | 71.70 (16.50) | 0.107 |
| AST | (U/L) | 31.30 (22.89) | 28.33 (22.73) | 0.130 |
| ALT | (U/L) | 28.01 (14.72) | 27.59 (15.89) | 0.028 |
| LDL-cholesterol | (mg/dL) | 129.83 (33.41) | 126.57 (32.20) | 0.099 |
| Triglycerides | (mg/dL) | 156.60 (126.64) | 142.49 (100.29) | 0.123 |
| Uric acid | (mg/dL) | 5.35 (1.30) | 5.24 (1.29) | 0.086 |

ALT, alanine aminotransferase; AST, aspartate aminotransferase; BMI, body mass index; DPP-4, dipeptidyl peptidase 4; GFR, glomerular filtration rate; GGT, gamma-glutamyl transpeptidase; HbA1c, hemoglobin A1c; LDL, low-density lipoprotein; SMD: standardized mean difference.
