# Supplementary material for: Comparing the effects of biguanides and dipeptidyl peptidase-4 inhibitors on cardio-cerebrovascular outcomes, nephropathy, retinopathy, neuropathy, and treatment costs in diabetic patients
Source: PLoS One. 2024 Aug 9;19(8):e0308734. doi: 10.1371/journal.pone.0308734 (PMC11315305; doi:10.1371/journal.pone.0308734)
Supplement: S6 Table — DPP-4: dipeptidyl peptidase-4; GLP-1: glucagon-like peptide-1; SGLT2: sodium–glucose cotransporter 2. (DOCX) [file pone.0308734.s006.docx]

**S6 Table.** Antidiabetic medication prescribed and the number of health care visits within the year following the index date, before propensity score matching.

| **Variable** | **Before matching** | |
| --- | --- | --- |
|  | **Biguanide**  **(n = 689)** | **DPP-4 inhibitor**  **(n = 3,625)** |
| Biguanides | 689 (100.0) | 0 |
| DPP-4 inhibitors | 0 | 3,625 (100.0) |
| Insulin | 0 | 0 |
| GLP-1 receptor agonists | 4 (0.6) | 2 (0.1) |
| SGLT2 inhibitors | 71 (10.3) | 144 (4.0) |
| Alpha-glucosidase inhibitors | 57 (8.3) | 279 (7.7) |
| Thiazolidinediones (also known as glitazones) | 16 (2.3) | 98 (2.7) |
| Rapid-acting secretagogues  (meglitinides, also known as glinides) | 34 (4.9) | 115 (3.2) |
| Sulfonylureas | 68 (9.9) | 406 (11.2) |
| Number of healthcare visits per year | 10.6±1.9 | 10.8±1.7 |

DPP-4: dipeptidyl peptidase-4; GLP-1: glucagon-like peptide-1; SGLT2: sodium-glucose cotransporter 2.
