# Supplementary material for: Comparing the effects of biguanides and dipeptidyl peptidase-4 inhibitors on cardio-cerebrovascular outcomes, nephropathy, retinopathy, neuropathy, and treatment costs in diabetic patients
Source: PLoS One. 2024 Aug 9;19(8):e0308734. doi: 10.1371/journal.pone.0308734 (PMC11315305; doi:10.1371/journal.pone.0308734)
Supplement: S7 Table — *Gray’s test was performed. †The log-rank test was performed. DPP-4: dipeptidyl peptidase 4 inhibitor. (DOCX) [file pone.0308734.s007.docx]

**S7** **Table.** Outcomes of the participants who were prescribed biguanide or a DPP-4 inhibitor in the matched cohort and who attended the clinic for ≥9 months, as a sensitivity analysis (n=2,634).

| **Outcome** | **Exposure** | **Events, number (%)** | **Cumulative incidence after 5 years** | | ***P*-value** |
| --- | --- | --- | --- | --- | --- |
|  |  |  | **Rate** | **95% Confidence interval** |  |
| Composite event**^†^** | Biguanide (n = 439) | 46 (10.2) | 11.5 | 8.3-15.9 | 0.395 |
|  | DPP-4 inhibitor (n = 2,195) | 224 (10.5) | 10.6 | 9.2-12.3 |  |
| Cardiac event^*^ | Biguanide (n = 439) | 22 (5.0) | 5.7 | 3.4-8.8 | 0.960 |
|  | DPP-4 inhibitor (n = 2,195) | 121 (5.5) | 5.7 | 4.6-7.0 |  |
| Cerebrovascular event^*^ | Biguanide (n = 439) | 15 (3.4) | 4.0 | 2.2-6.7 | 0.559 |
|  | DPP-4 inhibitor (n = 2,195) | 72 (3.3) | 3.3 | 2.5-4.3 |  |
| Death**^†^** | Biguanide (n = 439) | 16 (3.6) | 3.3 | 1.7 - 6.4 | 0.770 |
|  | DPP-4 inhibitor (n = 2,195) | 82 (3.7) | 3.3 | 2.5 - 4.4 |  |
| Diabetic complication^*^ | Biguanide (n = 439) | 74 (16.9) | 23.8 | 18.7-29.2 | 0.722 |
|  | DPP-4 inhibitor (n = 2,195) | 437 (19.9) | 23.0 | 20.9-25.2 |  |
| Diabetic retinopathy^*^ | Biguanide (n = 439) | 56 (12.8) | 17.0 | 12.8 – 21.8 | 0.973 |
|  | DPP-4 inhibitor (n = 2,195) | 319 (14.5) | 16.3 | 14.4 – 18.2 |  |
| Diabetic nephropathy^*^ | Biguanide (n = 439) | 20 (4.6) | 6.3 | 3.9 – 9.6 | 0.666 |
|  | DPP-4 inhibitor (n = 2,195) | 98 (4.5) | 4.7 | 3.7 – 5.8 |  |
| Diabetic neuropathy^*^ | Biguanide (n = 439) | 7 (1.6) | 2.2 | 0.9 – 4.6 | 0.606 |
|  | DPP-4 inhibitor (n = 2,195) | 47 (2.1) | 1.9 | 1.3 – 2.6 |  |
| Other conditions^*^ | Biguanide (n = 439) | 12 (2.7) | 2.6 | 1.2 – 4.9 | 0.991 |
|  | DPP-4 inhibitor (n = 2,195) | 67 (3.0) | 3.3 | 2.5 – 4.3 |  |

^*^Gray’s test was performed. ^†^The log-rank test was performed. DPP-4: dipeptidyl peptidase 4 inhibitor.
