# Supplementary material for: Comparing the effects of biguanides and dipeptidyl peptidase-4 inhibitors on cardio-cerebrovascular outcomes, nephropathy, retinopathy, neuropathy, and treatment costs in diabetic patients
Source: PLoS One. 2024 Aug 9;19(8):e0308734. doi: 10.1371/journal.pone.0308734 (PMC11315305; doi:10.1371/journal.pone.0308734)
Supplement: S8 Table — *Gray’s test was performed. †The log-rank test was performed. DPP-4: dipeptidyl peptidase 4 inhibitor. (DOCX) [file pone.0308734.s008.docx]

**S8** **Table.** Outcomes of participants who were prescribed biguanide or a DPP-4 inhibitor in the matched cohort and who had attended the clinic for ≥12 months, as a sensitivity analysis (n=1,602).

| **Outcome** | **Exposure** | **Events (%)** | **Cumulative incidence after 5 years** | | ***P*-value** |
| --- | --- | --- | --- | --- | --- |
|  |  |  | **Rate** | **95% Confidence interval** |  |
| Composite event**^†^** | Biguanide (n = 267) | 30 (11.2) | 11.3 | 7.4-16.9 | 0.835 |
|  | DPP-4 inhibitor (n = 1,335) | 161 (12.1) | 12.5 | 10.6-14.8 |  |
| Cardiac event^*^ | Biguanide (n = 267) | 16 (6.0) | 6.9 | 3.8-11.3 | 0.759 |
|  | DPP-4 inhibitor (n = 1,335) | 92 (6.9) | 7.2 | 5.7-8.9 |  |
| Cerebrovascular event^*^ | Biguanide (n = 267) | 11 (4.1) | 4.5 | 2.2-8.3 | 0.462 |
|  | DPP-4 inhibitor (n = 1,335) | 53 (4.0) | 3.7 | 2.6-5.0 |  |
| Death**^†^** | Biguanide (n = 267) | 9 (3.4) | 1.7 | 0.5-5.2 | 0. 837 |
|  | DPP-4 inhibitor (n = 1,335) | 55 (4.1) | 4.2 | 3.1-5.7 |  |
| Diabetic complication^*^ | Biguanide (n = 267) | 43 (16.1) | 21.3 | 15.3-28.0 | 0.348 |
|  | DPP-4 inhibitor (n = 1,335) | 271 (20.3) | 23.8 | 21.1-26.6 |  |
| Diabetic retinopathy^*^ | Biguanide (n = 267) | 35 (13.1) | 15.4 | 10.4 – 21.2 | 0.810 |
|  | DPP-4 inhibitor (n = 1,335) | 206 (15.4) | 17.2 | 14.9 – 19.7 |  |
| Diabetic nephropathy^*^ | Biguanide (n = 267) | 14 (5.2) | 6.8 | 3.7 – 11.1 | 0.974 |
|  | DPP-4 inhibitor (n = 1,335) | 74 (5.5) | 5.8 | 4.5 – 7.4 |  |
| Diabetic neuropathy^*^ | Biguanide (n = 267) | 4 (1.5) | 2.3 | 0.7 – 5.5 | 0.814 |
|  | DPP-4 inhibitor (n = 1,335) | 25 (1.9) | 1.7 | 1.0 – 2.6 |  |
| Other conditions^*^ | Biguanide (n = 267) | 6 (2.2) | 1.5 | 0.4 – 4.0 | 0.594 |
|  | DPP-4 inhibitor (n = 1,335) | 42 (3.1) | 3.1 | 2.2 – 4.3 |  |

^*^Gray’s test was performed. ^†^The log-rank test was performed. DPP-4: dipeptidyl peptidase 4 inhibitor.
